# The American Medical Association

**Published:** 1897-07

**Authors:** 


### THE AMERICAN MEDICAL ASSOCIATION.

The recent very successful meeting of this Association in Philadelphia, in point of numbers and enthusiasm, has demonstrated very conclusively that the professional spirit in that body has not been lessened, but is ever increasing and tending to broader and more thorough work.

The writer enjoyed the privilege of attending the meetings of the Dental and Oral Section, and found a body of men earnest and more intent upon the advancement of the scientific side of dentistry than they were of personal interests. This self-sacrificing devotion might, with great advantage, be manifested in other and more pretentious bodies.

The work of this section comprised many valuable papers, several of which seemed to attract the attention of the city press, large space being given especially to Dr. R. R. Andrews's views upon the dangers of tuberculosis through neglect of the teeth. If the section had done nothing more, the awakening of the general mind to this ever-present danger would have alone justified the meeting.

The attendance at the meeting of this section was not large, a fact to be regretted. This can, possibly, be accounted for by the general impression that membership is supposed to be confined to those having the medical degree, and therefore the mass of practitioners feel that they are not in touch with the section, however interested they may be in its work. The time has not yet arrived for the dental profession to closely affiliate with the medical, and this for many well-understood reasons not necessary to enlarge upon here. The fact remains, however, that this section, while composed of a body of strong men, cannot, as at present arranged, be a large organization or exercise the influence it should upon dentistry. It is possible, through a more liberal construction of its rules, to have this prejudice modified or removed altogether, but this must be left for time to determine.

The general work of the American Medical Association seemed to the writer to partake of the many objectionable features of all such organizations, including those of our own. The bringing together of some two thousand men in one body is not conducive to the best conditions, and the meetings at the Academy of Music were practically unmanageable. This was not wholly the fault of the president, whose voice was not equal to filling the large building, but, apparently, to a lack of adherence to parliamentary rules, fatal to all large meetings. This was peculiarly noticeable whenever a question deeply affecting the interests of the body was up for consideration, and then the gathering presented more the character of a tumultuous political meeting than of a grave scientific body.

The impression received was that, while the work of the Association was admittedly of great value, much was lost by a subdivision into sections, as the members could not attend them all, and they returned to their homes with a very indefinite idea of what had been accomplished outside of their own specialty. This seems to be a grave defect, not compensated for by the privilege of reading the papers as they are published in the *Journal.*

It was demonstrated, in the opinion of the writer, that this national medical organization has nearly reached a period in its history when its methods of work will require modification, if not an entire change in procedure, and this is equally applicable to dentistry. It is doubtful whether this will be recognized as a fact by the general medical or dental mind, but it must be conceded that the best scientific work cannot find a congenial atmosphere in great numbers, or in movable organizations. Time will bring about its changes, and to this may safely be left the future solution of the problem, and that with the assured consciousness that, however popular present methods may be, there will come a period when these will be regarded as antiquated and valueless as a means of bringing forth the best work.

